# Supplementary material for: Chiral phase-imaging meta-sensors
Source: Nanophotonics. 2025 Mar 25;14(7):947–57. doi: 10.1515/nanoph-2024-0759 (PMC11980873; doi:10.1515/nanoph-2024-0759)
Supplement: Supplementary file 1 — Supplementary Material Details [file j_nanoph-2024-0759_suppl_001.pdf]

Supplementary Material

Chiral phase-imaging meta-sensors

Ahmet M. Erturan†, Jianing Liu†, Maliheh A. Roueini†, Nicolas Malamug,

Lei Tian, and Roberto Paiella*

Department of Electrical and Computer Engineering and Photonics Center,

Boston University, 8 Saint Mary's Street, Boston, MA 02215

† These authors contributed equally

* rpaiella@bu.edu

S1. Meta-unit design

The detailed design simulations of the meta-units used in this work are summarized in Figs. S1(a) and S1(b), which show, respectively, the meta-unit reflection amplitude $|r_\xi|$ and phase $\arg\{r_\xi\}$ versus nanoparticle (NP) lateral dimensions L_ξ and L_ψ. These plots were computed via finite difference time domain (FDTD) simulations of a single meta-unit using periodic boundary conditions and illumination at normal incidence with linear polarization along the NP horizontal axis [the ξ direction in Fig. 2(a) of the main text with the angle α set equal to 0]. As expected, $\arg\{r_\xi\}$ can be tuned across the entire 2π phase range, while maintaining relatively large values of $|r_\xi|$. Identical plots with the L_ξ and L_ψ axes interchanged are obtained for the reflection phase and amplitude $\arg\{r_\psi\}$ and $|r_\psi|$ under linearly polarized light along the NP vertical axis (the ψ direction). These simulation results are combined in Fig. 2(b) of the main text, where we plot the scattering efficiency $\eta = \frac{1}{4}|r_\xi - r_\psi|^2$ versus $\varphi_{res} = \arg\{r_\xi - r_\psi\}$ for all simulated values of L_ξ and L_ψ (ranging from 0 to 600 nm in steps of 10 nm). The red circles in Figs. S1(a) and S1(b) indicate the NPs used in the 25 meta-units of both devices R and L described in the main text. These NPs were selected to produce the required values of the resonance phase φ_{res} while at the same time maximizing the scattering efficiency η. The lateral dimensions of all 25 NPs are listed in Table S1, together with their calculated values of η and φ_{res}.

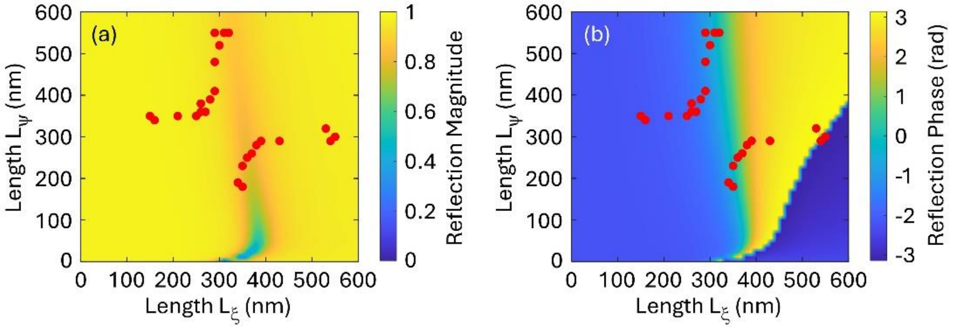

Figure S1. Calculated reflection amplitude $|r_\xi|$ (a) and phase $\arg\{r_\xi\}$ (b) of a single repeat unit of the metasurfaces developed in this work, plotted as a function of NP lateral dimensions. The red circles indicate the NPs used in the 25 meta-units of both devices R and L described in the main text.

Nanoparticle	L_ξ (nm)	L_ψ (nm)	Scattering Efficiency η	Resonance Phase φ_{res} (rad)
1	260	360	0.801	-1.374
2	290	550	0.840	-0.055
3	350	230	0.784	1.363
4	430	290	0.823	2.737
5	150	350	0.721	-2.146
6	280	390	0.804	-0.805
7	320	550	0.650	0.528
8	370	260	0.774	1.919
9	550	300	0.841	-3.002
10	250	350	0.791	-1.650
11	290	480	0.829	-0.215
12	350	180	0.751	1.126
13	390	290	0.823	2.483
14	160	340	0.649	-2.411
15	260	380	0.741	-1.106
16	310	550	0.771	0.340
17	360	250	0.786	1.691
18	540	290	0.838	3.070
19	210	350	0.772	-1.881
20	290	410	0.821	-0.513
21	340	190	0.699	0.884
22	380	280	0.810	2.242
23	530	320	0.642	-2.644
24	270	360	0.807	-1.280
25	300	520	0.835	0.083

Table S1. Lateral dimensions L_ξ and L_ψ, scattering efficiency $\eta = \frac{1}{4}\left|r_\xi - r_\psi\right|^2$, and resonance phase $\varphi_{res} = \arg\{r_\xi - r_\psi\}$ of all 25 NPs used in devices R and L. The NP ordering in the table is the same as in the device metasurfaces from left to right.

S2. Schematic device diagram

Figure S2 shows schematic top- and side-view diagrams of a complete device, showing all the dielectric and metallic layers deposited on the Ge substrate and their patterned shapes. The thin (10-nm) Ti films between the Ge substrate and the SiO_2 layer underneath the wire bonding pads are used to enhance the SiO_2/Ge adhesion. Similarly, the Au film and Au NPs include a 5-nm-thick Ti bottom adhesion layer (not included in these diagrams). The target layer thicknesses are 60/100/60 nm for the SiO_2/Au/SiO_2 stack supporting the NP array, and 50 nm for the Au NPs. The

slits (shown in the top-view diagram) are perforated through the entire stack. The patterned Ti window on the device top surface (120-nm thick) is used to suppress any spurious photocurrent that may otherwise be caused by light absorbed between the electrodes away from the metasurface.

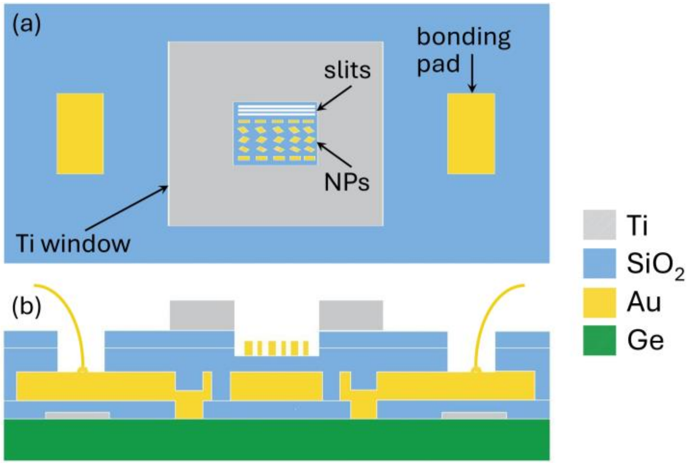

Figure S2. Schematic top (a) and side (b) views of a complete device, including the Au bond wires for biasing and photocurrent collection.

S3. First-order diffraction peak

In the metasurfaces of both devices R and L, the NPs are arranged periodically in a square lattice. As a result, despite their different dimensions and orientations, they can diffract the incident light. In particular, as shown by the simulation results of Fig. 1(b) and the experimental data of Figs. 3(d) and 3(g), an additional peak at $\theta \approx 22°$ is introduced in the device angular response via negative first-order diffraction. The physical origin of this peak is illustrated in Fig. S3(a): through the combined action of the metasurface linear phase profile and diffraction, the incident-light wavevector is shifted by $\hat{\mathbf{x}}(\Delta k_{tot} - 2\pi/\Delta x)$ and as a result surface plasmon polaritons (SPPs) propagating in the negative x direction are excited. Here, the x direction is as defined in Fig. 1(a), Δk_{tot} is the slope of the metasurface reflection phase versus x, and Δx = 600 nm is the NP array period. In our simulated and experimental devices, the NP arrays are surrounded by slits on both sides. Thus, the SPPs excited through the process of Fig. S3(a) are eventually intercepted by the slits on the left-hand side (i.e., in the negative x direction) and scattered into the photodetector active layer. It should also be noted that, for the specific values of all the geometrical parameters and operation wavelength of these metasurfaces, no other nonzero diffraction order can couple light incident at any angle into either radiation modes or SPPs. The main radiative channel for the

incident light is spurious specular reflection by zero-order diffraction, and a total reflection coefficient of about 46% has been computed for devices R and L at their angle and polarization of peak detection, which represents a sizeable fraction of the overall losses of these devices.

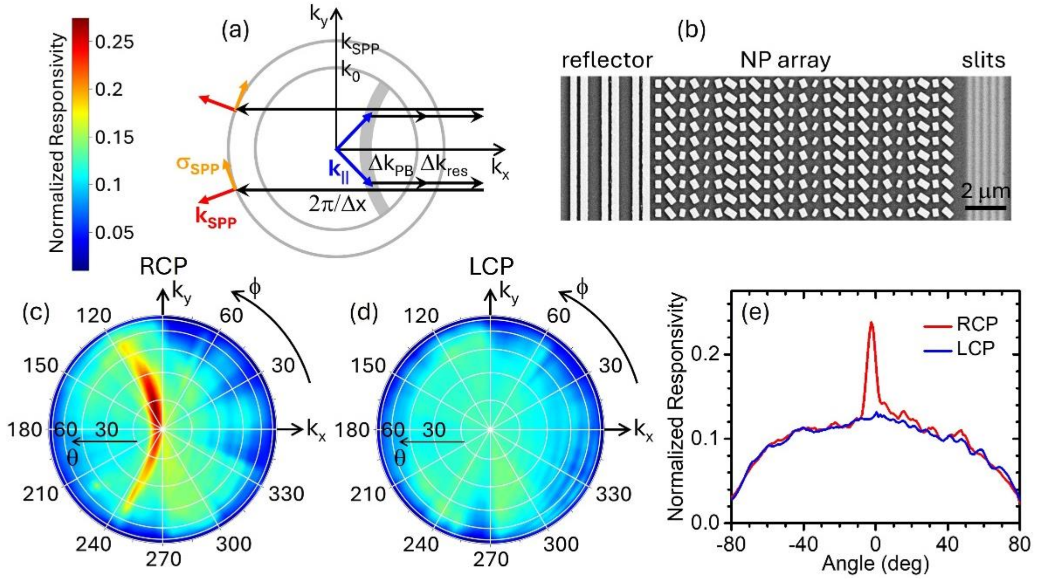

Figure S3. (a) Reciprocal-space diagram illustrating the plasmon excitation process responsible for the first-order diffraction peak observed in the angular response of devices R and L. The blue arrows indicate the in-plane wavevectors $\mathbf{k}_{||}$ of light incident along two representative directions. The red and orange arrows represent the wavevector $\mathbf{k}_{SPP}$ and spin angular momentum $\boldsymbol{\sigma}_{SPP}$ of the excited SPPs. The combined phase-matching action of the NP-array diffraction and the metasurface resonance and PB phase is indicated by the horizontal black arrows. (b) Top-view SEM image of a variation of device R designed to suppress the first-order diffraction peak. (c), (d) Normalized responsivity of this device measured as a function of polar θ and azimuthal ϕ angles of incidence for RCP (c) and LCP (d) light. (e) Horizontal line cuts of the color maps of (c) and (d) (red and blue traces, respectively).

For the phase imaging applications considered in this work, the imaging optics limits the angles of incidence on the sensor array to small values of a few degrees. As a result, the additional peak at $\theta \approx 22°$ caused by negative-first-order diffraction is inconsequential. At the same time, for other applications of directional photodetectors such as lensless compound-eye vision [S1], the ability to synthesize a single-peak angular response can be desirable. In the present devices, this goal can be accomplished simply by removing the slits on the left-hand side of the NPs. To avoid possible interpixel crosstalk in a sensor array, these slits can be replaced with an additional metasurface section (reflector) designed to scatter all incoming SPPs into free-space radiation propagating away from the device surface. A scanning electron microscopy (SEM) image of a variation of device R based on this approach is shown in Fig. S3(b). The reflector consists of multiple Au nanostripes of different widths selected to produce the required phase slope for

efficient SPP scattering. Its design is described in detail in ref. S1, where it was used to produce an asymmetric angular response in conjunction with a diffraction grating.

The measured angular response of this device is shown in Figs. S3(c)-(e). As expected, these data are analogous to those measured with device R [Figs. 3(b)-(d)], without the additional responsivity peak originating from negative-first-order diffraction. The reflector section has a total width of 7.6 μm, which adds to the combined width of 17 μm for the NP array and the slit section on its right-hand side. Therefore, removal of the diffraction peak to produce a single-peak angular response comes at the expense of an increase in the device footprint, which may limit the spatial resolution for imaging with a pixel array of these photodetectors.

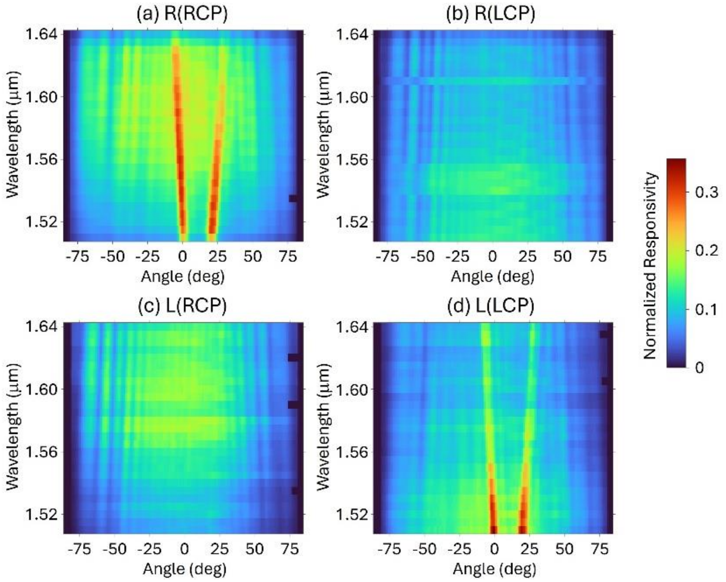

Figure S4. (a), (b) Normalized responsivity of device R measured as a function of angle of incidence θ (for $k_y = 0$) and wavelength under RCP (a) and LCP (b) illumination. (c), (d) Same as (a) and (b) for device L.

S4. Wavelength dependence of the angular response

Figures S4(a) and S4(b) show, respectively, the normalized RCP and LCP responsivity of device R measured as a function of angle of incidence (for $k_y = 0$) at different wavelengths from 1510 to 1640 nm. A steady shift in the two responsivity peaks with wavelength is clearly observed in the RCP map, as expected from the phase-matching condition discussed in the main text. Furthermore, as the wavelength is detuned away from its design value of 1550 nm, the strength of both peaks eventually begins to decrease, due to increasing deviations in the metasurface resonance phase $\varphi_{\mathrm{res}}(x)$ from a linear profile. The same behavior is observed in the wavelength

dependence of the angular response of device L [Figs. S4(c) and S4(d)]. These data were used to determine the normal-incidence responsivity slopes as a function of wavelength plotted in Fig. 4 of the main text.

S5. Transfer-function phase fitting

The phase imaging simulations presented in the main text are based on the model of eq. (3), which requires knowledge of the angle-resolved RCP and LCP responsivities $\mathcal{R}_{\mathrm{RCP}}(\mathbf{k})$ and $\mathcal{R}_{\mathrm{LCP}}(\mathbf{k})$ and of the phase difference $\Delta(\mathbf{k})$ between the RCP and LCP transfer functions. The measured responsivity maps $\mathcal{R}_{\mathrm{RCP}}(\mathbf{k})$ and $\mathcal{R}_{\mathrm{LCP}}(\mathbf{k})$ used in these simulations are shown in Fig. 3. Here we discuss how the phase difference $\Delta(\mathbf{k})$ can be determined from additional angle-resolved photocurrent measurements with linearly polarized light.

According to eq. (3), under plane-wave illumination with in-plane wavevector $\mathbf{k}$ and arbitrary polarization, the responsivity of these devices can be expressed as

$$\mathcal{R}(\mathbf{k}) = \left|\sqrt{\mathcal{R}_{\mathrm{RCP}}(\mathbf{k})}e^{i\Delta(\mathbf{k})}\tilde{E}_{\mathrm{RCP}} + \sqrt{\mathcal{R}_{\mathrm{LCP}}(\mathbf{k})}\tilde{E}_{\mathrm{LCP}}\right|^2, \tag{S1}$$

where $\tilde{E}_{\mathrm{RCP}}$ and $\tilde{E}_{\mathrm{LCP}}$ are the RCP and LCP components of the incident polarization unit vector. For the special cases of p and s polarizations, $\tilde{E}_{\mathrm{RCP}} = \pm\,\tilde{E}_{\mathrm{LCP}} = 1/\sqrt{2}$, and eq. (S1) becomes

$$\mathcal{R}_{p/s}(\mathbf{k}) = \left|\sqrt{\mathcal{R}_{\mathrm{RCP}}(\mathbf{k})}e^{i\Delta(\mathbf{k})} \pm \sqrt{\mathcal{R}_{\mathrm{LCP}}(\mathbf{k})}\right|^2/2. \tag{S2}$$

Using this formula, the phase difference $\Delta(\mathbf{k})$ could be extracted by fitting the p and s responsivity data with the RCP and LCP results.

This analysis applied to device R is shown in Fig. S5. The black traces in Fig. S5(a) show the measured p and s responsivities versus angle of incidence θ for $k_y = 0$ and $k_x = (2\pi/\lambda_0)\sin\theta$. The red traces were computed from eq. (S2) with k_x-dependent fitting parameter Δ, using the measured values of $\mathcal{R}_{\mathrm{RCP}}(k_x, k_y = 0)$ and $\mathcal{R}_{\mathrm{LCP}}(k_x, k_y = 0)$ from Fig. 3(d). Good agreement between the experimental and fitted data for both s and p polarization is obtained for the values of Δ plotted in Fig. S5(b). The two peaks observed in this trace are associated with the phase variations across the resonances responsible for the corresponding peaks in the responsivity data. Figures S5(c) and S5(d) show the results of the same analysis using the metasurface transmission coefficients computed as a function of illumination angle for all four polarizations with FDTD simulations (in these devices, the angular dependence of the responsivity is entirely determined by

the metasurface transmission). The similarity between the fitted traces of Δ versus θ based on the experimental and numerical data [Figs. S5(b) and S5(d), respectively] highlights the validity of this procedure. Analogous results were obtained for device L.

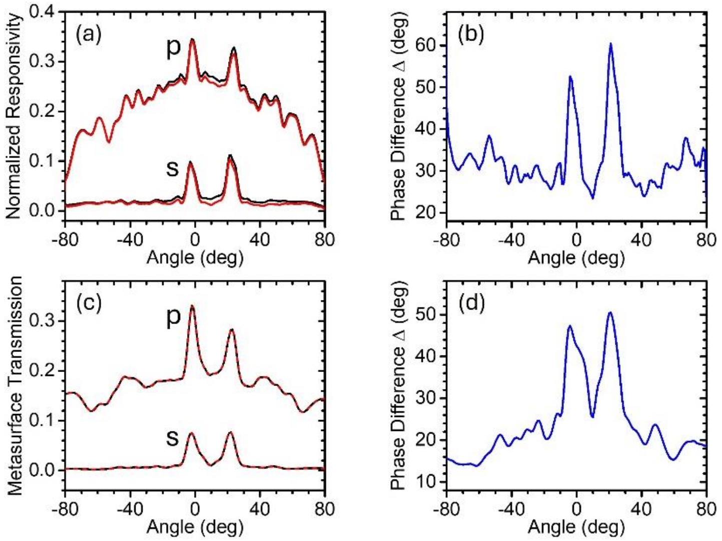

Figure S5. (a) Black traces: normalized responsivity of device R measured as a function of angle of incidence θ (for $k_y = 0$) under p and s polarized illumination. Red traces: numerical fits of the black traces based on the measured RCP and LCP angular responses of the same device using eq. (S2). (b) Phase difference $\Delta(\mathbf{k})$ between the RCP and LCP transfer functions of this device inferred from the fits shown in (a). (c), (d) Same as (a) and (b) using the calculated angle-dependent p, s, RCP, and LCP transmission coefficients of the metasurface of the same device.

In the imaging simulations presented in the main text, we approximate $\Delta(\mathbf{k})$ with its normal-incidence value $\Delta(0)$ = 43.5° from Fig. S5(b) [versus 42.6° in Fig. S5(d)]. This approximation is justified by the small range of incident angles relevant to these simulations (< 2.3°), and the proportionally small variations in $\Delta(\mathbf{k})$ across this range.

S6. Phase-image reconstruction protocol

The reconstructed phase distribution $\varphi_{\mathrm{RCP}}(\mathrm{x})$ shown in Fig. 6(c) of the main text was computed from the differential-phase-contrast image $\mathrm{S_R}(\mathrm{x})$ of Fig. 6(a) by solving the equation

$$\mathrm{S_R}(\mathbf{r}) = \iint \frac{\mathbf{dk}}{(2\pi)^2} \mathrm{H_R}(\mathbf{k}) \varphi_{\mathrm{RCP}}(\mathbf{k}) \mathrm{e}^{\mathbf{ik \cdot r}} , \tag{S3}$$

with

$$\mathrm{H_R}(\mathbf{k}) = i\mathrm{t}_{\mathrm{lens}}(\mathbf{k})\mathrm{Re}\left\{e^{i\Delta(\mathbf{k})}\sqrt{\mathcal{R}_{\mathrm{RCP}}(\mathbf{k})}\Big/\left[e^{i\Delta(\mathbf{k})}\sqrt{\mathcal{R}_{\mathrm{RCP}}(0)} + \sqrt{\mathcal{R}_{\mathrm{LCP}}(0)}\right]\right\}. \tag{S4}$$

Here, $t_{lens}(\mathbf{k})$ is the pupil function of the imaging system in front of the sensor array (i.e., a cylindrical step function with cutoff frequency $k_c = 2\pi NA/\lambda_0$), $\mathcal{R}_{RCP}(\mathbf{k})$ and $\mathcal{R}_{LCP}(\mathbf{k})$ are the RCP and LCP responsivity functions of device R, and $\Delta(\mathbf{k})$ is the phase factor just described. In this section, we outline the derivation of eqs. (S3) and (S4). Similar considerations (with RCP and LCP interchanged, and using the responsivity functions of device L) apply to the reconstruction of the phase distribution $\varphi_{LCP}(x)$ from $S_L(x)$.

In eq. (S3), $S_R = (I_{\bar{R}} - I_R)/(I_{\bar{R}} + I_R)$, where I_R and $I_{\bar{R}}$ are the photocurrent signals measured by device R and its rotated replica $\bar{R}$ in each superpixel as a function of the superpixel position **r** within the sensor array. From eq. (3) of the main text,

$$I_R(\mathbf{r}) = C_0 \iint \frac{d\mathbf{k}}{(2\pi)^2} \left\{ \sqrt{\mathcal{R}_{RCP}(\mathbf{k})} e^{i\Delta(\mathbf{k})} E_{RCP}(\mathbf{k}) + \sqrt{\mathcal{R}_{LCP}(\mathbf{k})} E_{LCP}(\mathbf{k}) \right\} e^{i\mathbf{k}\cdot\mathbf{r}}$$

$$\times \iint \frac{d\mathbf{k}'}{(2\pi)^2} \left\{ \sqrt{\mathcal{R}_{RCP}(\mathbf{k}')} e^{-i\Delta(\mathbf{k}')} E^*_{RCP}(\mathbf{k}') + \sqrt{\mathcal{R}_{LCP}(\mathbf{k}')} E^*_{LCP}(\mathbf{k}') \right\} e^{-i\mathbf{k}'\cdot\mathbf{r}}, \qquad \text{(S5)}$$

where C_0 is a constant. A similar expression applies for $I_{\bar{R}}(\mathbf{r})$ with $\mathcal{R}_X(\mathbf{k})$ replaced by $\mathcal{R}_X(-\mathbf{k})$, where X = RCP or LCP.

In this derivation, we consider the specific situation where a circularly birefringent pure phase object is illuminated with a linearly polarized plane wave. After transmission through the object, the circular polarization components of the optical field can then be written as $E_X(\mathbf{r}) = \sqrt{P} e^{i\varphi_X(\mathbf{r})}$, where P is a constant power and $\varphi_X(\mathbf{r})$ indicates the X-polarized phase shift induced by the object. In the weak-object approximation [S2], $E_X(\mathbf{r})$ can be linearized with respect to $\varphi_X(\mathbf{r})$, and its Fourier transform becomes

$$E_X(\mathbf{k}) \approx \sqrt{P}[(2\pi)^2 \delta(\mathbf{k}) + i\varphi_X(\mathbf{k})], \qquad \text{(S6)}$$

where $\delta(\mathbf{k})$ is the delta function and $\varphi_X(\mathbf{k})$ is the Fourier transform of $\varphi_X(\mathbf{r})$.

Next, we substitute eq. (S6) into eq. (S5) and neglect all terms proportional to products of the small quantities $\varphi_X(\mathbf{k})$ and $\varphi_X(\mathbf{k}')$. Additionally, we approximate $\mathcal{R}_{LCP}(\mathbf{k})$ with $\mathcal{R}_{LCP}(0)$, consistent with the nearly constant responsivity of device R with respect to the angle of incidence of the LCP component [see Fig. 3(c)]. Correspondingly, the terms proportional to $\varphi_{LCP}(\mathbf{k})$ and $\varphi_{LCP}(\mathbf{k}')$ in eq. (S5) cancel each other, so that $I_R(\mathbf{r})$ does not depend on $\varphi_{LCP}(\mathbf{r})$ as expected. Finally, we use the resulting expressions for $I_R(\mathbf{r})$ and $I_{\bar{R}}(\mathbf{r})$ to compute $S_R(\mathbf{r})$, leading to eqs. (S3) and (S4).

These equations can be used to reconstruct any phase object $\varphi_{RCP}(\mathbf{r})$ from its measured differential-phase-contrast image $S_R(\mathbf{r})$, as we have done in Fig. 6(c) of the main text. Importantly, in these calculations we have also added Gaussian noise to each image to account for the noise properties of image-sensor photodiodes [S3, S4]. To avoid numerical issues associated with noise amplification, instead of direct inversion of eq. (S3) we have then used the following regularized least-squares minimization

$$\min\{|S_R(\mathbf{k}) - H_R(\mathbf{k})\varphi_{RCP}(\mathbf{k})|^2 + \alpha_T|\varphi_{RCP}(\mathbf{k})|^2\}, \quad \text{(S7)}$$

where α_T is a regularization parameter. The solution of this minimization problem is known as Tikhonov regularization [S2] and is given as follows:

$$\varphi_{RCP}(\mathbf{r}) = \mathcal{F}^{-1}\left\{\frac{H_R^*(\mathbf{k})S_R(\mathbf{k})}{|H_R(\mathbf{k})|^2+\alpha_T}\right\}, \quad \text{(S8)}$$

where $\mathcal{F}^{-1}$ indicates the inverse Fourier transform.

A similar derivation can be extended to the more general case of an object that exhibits both circular birefringence and circular dichroism.

Supplementary References

S1. L. C. Kogos, Y. Li, J. Liu, Y. Li, L. Tian, and R. Paiella, "Plasmonic ommatidia for lensless compound-eye vision," *Nat. Commun.*, vol. 11, art. no. 1637, 2020.

S2. L. Tian and L. Waller, "Quantitative differential phase contrast imaging in an LED array microscope," *Opt. Express.*, vol. 23, pp. 11394−11403, 2015.

S3. J. Liu, H. Wang, Y. Li, L. Tian, and R. Paiella, "Asymmetric metasurface photodetectors for single-shot quantitative phase imaging," *Nanophotonics*, vol. 12, pp. 3519-3528, 2023.

S4. M. Murata, R. Kuroda, Y. Fujihara, Y. Otsuka, H. Shibata, T. Shibaguchi, Y. Kamata, N. Miura, N. Kuriyama, and S. Sugawa, "A high near-infrared sensitivity over 70-dB SNR CMOS image sensor with lateral overflow integration trench capacitor," *IEEE Trans. Electron Devices*, vol. 67, pp. 1653-1659, 2020.
